# Higher anticholinergic burden from medications is associated with significant increase in markers of inflammation in the EPIC‐Norfolk prospective population‐based cohort study

**DOI:** 10.1111/bcp.15261

**Published:** 2022-02-27

**Authors:** Ria Sanghavi, Tiberiu A. Pana, Hulkar Mamayusupova, Ian Maidment, Chris Fox, S. Matthijs Boekholdt, Mamas A. Mamas, Nicholas J. Wareham, Kay‐Tee Khaw, Phyo K. Myint

**Affiliations:** ^1^ College of Life Sciences University of Leicester UK; ^2^ Aberdeen Diabetes and Cardiovascular Centre, School of Medicine, Medical Sciences & Nutrition University of Aberdeen Aberdeen Scotland UK; ^3^ Ageing Clinical and Experimental Research Team University of Aberdeen UK; ^4^ Norwich Medical School University of East Anglia Norwich UK; ^5^ Pharmaceutical & Clinical Pharmacy Research Group Aston University Birmingham UK; ^6^ Department of Cardiology Amsterdam University Medical Centre, location AMC Amsterdam The Netherlands; ^7^ Keele Cardiovascular Research Group Keele University Stoke‐on‐Trent UK; ^8^ MRC Epidemiology Unit Cambridge UK; ^9^ Clinical Gerontology Unit, Department of Public Health & Primary Care University of Cambridge Cambridge UK

**Keywords:** anticholinergics, cardiovascular diseases, C‐reactive protein, fibrinogen, interleukin‐6, tumour necrosis factor‐alpha

## Abstract

**Background:**

Higher medication anticholinergic burden is associated with increased risk of cardiovascular disease and cognitive decline. A mechanistic pathway has not been established. We aimed to determine whether inflammation may mediate these associations.

**Methods:**

Participants were drawn from the European Prospective Investigation into Cancer, Norfolk cohort (40‐79 years at baseline). Anticholinergic burden score (ACB) was calculated at first (1HC) (1993/97) and second (2HC) (1998/2000) health checks. Fibrinogen and C‐reactive protein (CRP) were measured during 1HC and tumour necrosis factor alpha (TNF‐α) and interleukin 6 (IL‐6) during 2HC. Cross‐sectional associations between ACB and inflammatory markers were examined for both health checks. Prospective associations were also examined between 1HC ACB and 2HC inflammatory markers. Models were adjusted for age, sex, lifestyle factors, comorbidities and medications.

**Results:**

In total, 17 678 and 22 051 participants were included in cross‐sectional analyses for CRP, and fibrinogen, respectively. Furthermore, 5101 participants with data on TNF‐α and IL‐6 were included in the prospective analyses. Cross‐sectionally, compared to ACB = 0, ACB ≥ 4 was associated with higher fibrinogen, beta (95% confidence interval) = 0.134 g/L (0.070, 0.199), CRP 1.175 mg/L (0.715, 1.634), IL‐6 0.593 pg/mL (0.254, 0.932) and TNF‐α 0.137 pg/mL (0.033, 0.241). In addition, a point increase in ACB was associated with higher levels of all markers. Prospectively, compared to ACB = 0, ACB ≥ 4 was associated with higher IL‐6(pg/mL) of 0.019 (−0.323, 0.361) and TNF‐α (pg/mL) of 0.202% (0.81, 0.323). A unit increase in ACB was associated with a significantly higher TNF‐α and IL‐6.

**Conclusion:**

Higher ACB was associated with higher inflammatory markers. Inflammation may mediate the relationship between anticholinergic medications and adverse outcomes.

**What is known about this subject**

- Higher anticholinergic burden from medications is associated with increased risk of cardiovascular disease and cognitive function decline.

**What this study adds**

- Using data from a large‐scale prospective cohort study from UK, we determined the cross‐sectional and prospective associations between the anticholinergic burden and inflammatory markers.
- Inflammation may mediate the relationship between exposure to anticholinergic medications and adverse outcomes through the “inflammatory reflex” or cholinergic anti‐inflammatory pathway.

## 1 INTRODUCTION

Anticholinergic medications block the effect of the neurotransmitter acetylcholine by inhibiting muscarinic receptors^1^ and are increasingly being prescribed to older people for common conditions including asthma, urinary incontinence and dementia.^2^ Anticholinergic effects are present in drugs that are extensively used, such as antiemetics, antihistamines, antihypertensives and tricyclic antidepressants.^3^ If one or more of such medications are taken, they can cause excessive anticholinergic effects due to their cumulative effects, a phenomenon also known as anticholinergic burden.^4^

Older people are at a particularly higher risk of anticholinergic complications due to (a) increased risk of polypharmacy including medications with anticholinergic properties, (b) age‐related reductions in central cholinergic pathways and (c) decreases in the renal and hepatic clearance of drugs.^4^ It was suggested that 48% of the ageing population may take one or more anticholinergic medications.^5^ Higher anticholinergic burden (ACB) scores are also associated with an increased risk of cardiovascular disease and mortality,^6^, ^7^ dementia^8^ and adverse effects in cognitive and physical function.^9^ Nevertheless, potential mechanistic pathways between these associations have not been identified.

Inflammation plays an important role in the pathogenesis of cardiovascular disease^10^, ^11^, ^12^ and dementia.^13^ Raised inflammatory markers have also been linked to depression^14^ and functional decline in older people.^15^ The vagus nerve may mediate inflammation through the inflammatory reflex.^16^ Previous preclinical data suggest that central muscarinic‐dependent vagal activation contributes to the downregulation of inflammatory responses.^17^

We therefore hypothesised that increased anticholinergic burden leads to increased inflammation by blocking the central muscarinic‐dependent vagal activation. This may mediate the previously described relationships between anticholinergic burden and adverse outcomes. No previous investigations have assessed the relationship between antimuscarinic activity and inflammation in a population sample. In this study, we aimed to examine the cross‐sectional and prospective relationships between anticholinergic burden from medications and important inflammatory markers (plasma fibrinogen, C‐reactive protein [CRP], tumour necrosis factor alpha [TNF‐α] and interleukin 6 [IL‐6]) in a large UK population‐based study.

## 2 METHODS

### 2.1 Participants

**FIGURE 1:**
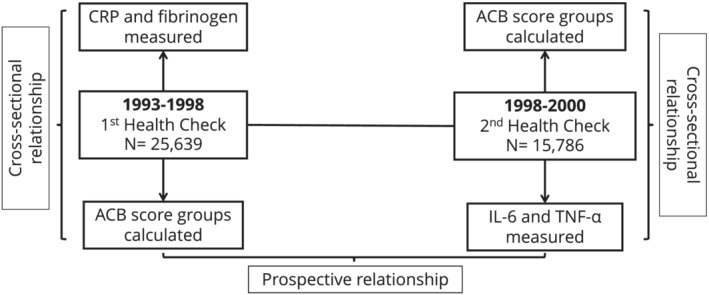
The EPIC‐Norfolk cohort timeline and the analyses undertaken

The participants were drawn from 25 639 men and women who enrolled in the EPIC‐Norfolk study at baseline (first health check, 1HC) between 1993 and 1998. The study protocol has been previously described.^18^ Study participants were followed up between 1998 and 2000, and 15 786 participants attended the second health check (2HC). The Norwich Research Ethics Committee approved the study. Figure 1 summarises the timeline of the EPIC‐Norfolk study and the analyses undertaken.

### 2.2 Measurements

During both health checks, trained nurses measured weight, height and body mass index (BMI) using standardized procedures. Participants also completed a health and lifestyle questionnaire that collected information on physical activity, smoking status, alcohol consumption, comorbid conditions and medications. The Food Frequency Questionnaire (FFQ) was used to measure participants' habitual dietary pattern during the previous year. The questionnaires were collected at the 1HC and after 5 years. Nonfasting venous blood samples were also taken. From each individual, ~40 mL of blood was drawn and serum, plasma, erythrocytes and buffy coat were aliquoted in plastic straws of 0.5 mL each. These straws were then heat‐sealed and stored under liquid nitrogen (−196 °C) in a centralized biobank. Figure 1 details the measurement of each inflammatory marker in relation to the study timeline: CRP and fibrinogen were measured at 1HC (1993‐1998), whilst TNF‐α and IL‐6 were measured at 2HC (1998‐2000).

### 2.3 Exposure

Medications with anticholinergic properties were identified by searching the database for exact and similar entries for both generic and brand name drugs. A corresponding anticholinergic class/score was allocated to each medication: class 0 (none), class 1 (probable, score 1), classes 2 and 3 (definite, score 2 and 3, respectively). This was based on the criteria of the anticholinergic cognitive burden (ACB) scale developed by Boustani et al.^19^ since this is one of the best‐known scales and validated against many clinical outcomes of interest. Subsequently, the total anticholinergic burden was then calculated for each participant by adding the individual ACB scores of all their medications at baseline. Participants were divided into six groups according to their total ACB score at baseline: 0, 1, 2, 3, 4, ≥5 (due to small sample size for ACB score 6 and above) for meaningful analysis.

### 2.4 Outcome measures

The inflammatory markers were the outcomes of interest: plasma fibrinogen, CRP, IL‐6 and TNF‐α. Fibrinogen (g/L) and CRP (mg/L) were available for a subsample of 1HC, and IL‐6 (pg/mL) and TNF‐α (pg/mL) were available for a subsample in 2HC.

### 2.5 Statistical analysis

Data were analysed using SPSS version 24.0 (SPSS Inc., Chicago, IL, USA). Descriptive statistics were presented for the overall sample and by ACB score groups. Differences between ACB groups were assessed using the Pearson's chi‐squared test for categorical variables and analysis of variance (ANOVA) for normally distributed continuous variables or the Kruskal‐Wallis test for continuous variables that were not normally distributed.

Linear regression analysis was performed to determine the cross‐sectional relationship of baseline/1HC ACB and 1HC inflammatory markers, fibrinogen and CRP. Linear regression was also performed to determine the cross‐sectional association of 2HC ACB and 2HC inflammatory markers IL‐6 and TNF‐α. The prospective relationship between baseline/1HC ACB data and 2HC inflammatory markers IL‐6 and TNF‐α was also determined.

Four statistical models were constructed to assess the effects of potential confounding factors in a group sequential fashion. Model A was unadjusted, model B was adjusted for age and sex, model C was adjusted for age, sex and lifestyle factors (BMI, alcohol consumption, smoking status, total fruits and vegetables consumed and physical activity) and, lastly, model D, the fully adjusted model, was adjusted for the variables age, sex, lifestyle factors, comorbidities (diabetes, stroke, cancer and heart attack), medications (lipid‐lowering drugs, nonsteroidal anti‐inflammatory drugs [NSAIDs]) and total cholesterol.

## 3 RESULTS

**FIGURE 2:**
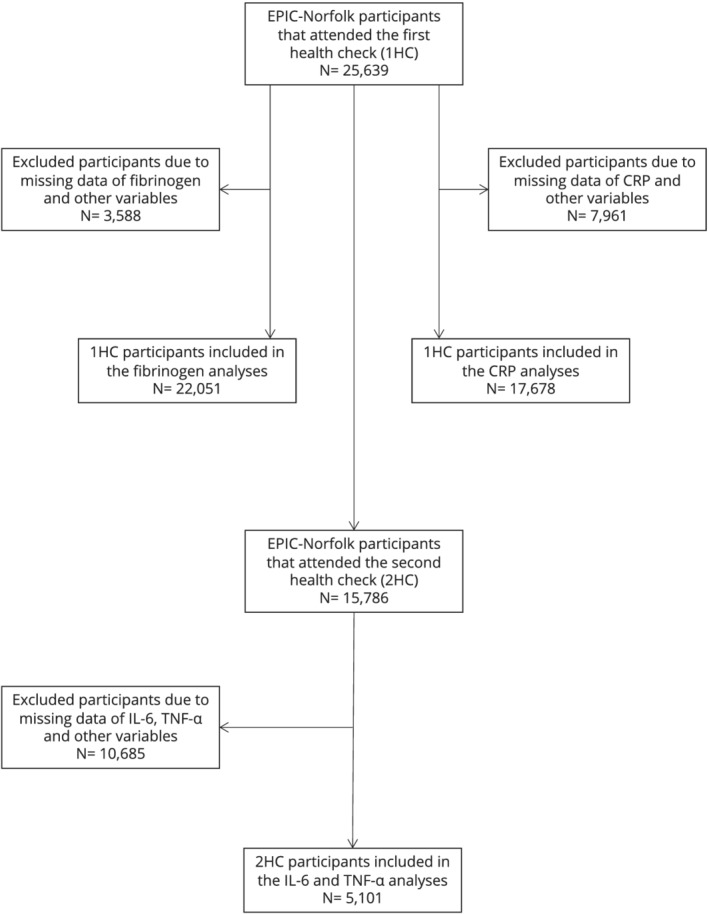
The participant population flowchart

There were 25 639 participants that attended the 1HC. A total of 3588 participants were excluded from the fibrinogen analysis and 7961 individuals were excluded from the CRP analysis due to missing data. Therefore, a total of 22 051 and 17 678 participants were included in the fibrinogen and CRP analyses, respectively. In the 2HC, out of the 15 786 participants who attended, only 5101 men and women were included in the analysis for TNF‐α and IL‐6 after the exclusion of 10 685 participants with missing data for these variables. Figure 2 displays the participant population flowchart.

All exclusions were made due to missing data. Participants that did not have a response (missing data) for diabetes mellitus, myocardial infarction, cerebrovascular accident, cancer and cholesterol were counted as not having the condition and were therefore not missing from the analysis. Similarly, those that did not respond for smoking status, alcohol consumption, NSAIDs and lipid‐lowering drugs were counted as not consuming these and therefore not missing from the analysis. In the 1HC, there were three participants with missing data for sex and 890 for fruit and vegetable consumption, whilst in the 2HC there were 1002 participants with missing data for physical activity and 3543 for fruit and vegetables.

TNF‐α and IL‐6 were analysed together as they had similar amounts of missing data.

### 3.1 Descriptive statistics

Tables 1 and Supporting Information Table S1 (online resources) detail the sample characteristics at baseline/1HC by ACB group. Table 1 details the descriptive characteristics of included participants in the fibrinogen analysis. The mean (SD) age was 59.1 years (9.3), with 54% being female. The mean (SD) plasma fibrinogen was 2.9 g/L (1.0). Supporting Information Table S1 details the descriptive statistics of included participants in the CRP analysis. The mean age (SD) was 59.1 years (9.1) for Supporting Information Table S1, with 55.1% being female. The median (IQR) CRP was 1.5 mg/L (0.7‐3.3).

**TABLE 1:** Baseline sample characteristics for 22 051 men and women whose fibrinogen level was measured in the EPIC‐Norfolk cohort (first health check) according to the total anticholinergic burden score groups

	All	ACB score 0 group	ACB score 1 group	ACB score 2 group	ACB score 3 group	ACB score 4 group	ACB score ≥5 group	*P* value
	n = 22 051	n = 17 609	n = 2252	n = 542	n = 677	n = 409	n = 562	
Mean age (years) (SD)	59.1 (9.3)	58.2 (9.1)	62.4 (8.9)	65.5 (8.3)	62.0 (9.2)	65.0 (7.9)	62.7 (9.0)	<.001
Sex (%)								<.001
Men	10 140 (46.0)	8065 (45.8)	1059 (47.0)	294 (54.2)	274 (40.5)	214 (52.3)	234 (41.6)	
Women	11 911 (54.0)	9544 (54.2)	1193 (53.0)	248 (45.8)	403 (59.5)	195 (47.7)	328 (58.4)	
Mean BMI (kg/m^2) (SD)	26.3 (3.8)	26.1 (3.7)	27.0 (4.2)	27.0 (4.0)	27.0 (4.1)	27.2 (4.2)	27.2 (4.1)	<.001
Smoking status (%)								<.001
Current	2470 (11.2)	2012 (11.4)	215 (9.5)	54 (10.0)	72 (10.6)	34 (8.3)	83 (14.8)	
Former	9294 (42.1)	7186 (40.8)	1066 (47.3)	276 (50.9)	309 (45.6)	206 (50.4)	251 (44.7)	
Never	10 287 (46.7)	8411 (47.8)	971 (43.1)	212 (39.1)	296 (43.7)	169 (41.3)	228 (40.6)	
Median alcohol consumption (units/week) (IQR)	3.5 (1.0‐10.0)	4.0 (1.5‐10.0)	2.5 (1.0‐9.0)	2.5 (1.0‐8.5)	2.5 (0.5‐8.5)	2.5 (0.5‐9.0)	2.0 (0.5‐7.0)	<.001
Physical activity (%)								<.001
Inactive	6527 (29.6)	4745 (26.9)	857 (38.1)	251 (46.3)	248 (36.6)	188 (46.0)	238 (42.3)	
Moderately inactive	6351 (28.8)	5112 (29.0)	637 (28.3)	130 (24.0)	200 (29.5)	109 (26.7)	163 (29.0)	
Moderately active	5088 (23.1)	4262 (24.2)	445 (19.8)	84 (15.5)	136 (20.1)	52 (12.7)	109 (19.4)	
Active	4085 (18.5)	3490 (19.8)	313 (13.9)	77 (14.2)	93 (13.7)	60 (14.7)	52 (9.3)	
Mean total cholesterol (mmol/L) (SD)	6.2 (1.2)	6.1 (1.1)	6.3 (1.2)	6.4 (1.3)	6.3 (1.2)	6.4 (1.3)	6.4 (1.3)	<.001
Mean total fruits and vegetables consumed (g/d) (SD)	511.3 (249.8)	509.8 (248.2)	518.8 (261.2)	504.7 (260.4)	524.0 (251.6)	514.3 (229.0)	517.6 (257.0)	.16
NSAID (%)	3266 (14.8)	2136 (12.1)	514 (22.8)	167 (30.8)	182 (26.9)	117 (28.6)	150 (26.7)	<.001
Lipid lowering drugs (%)	329 (1.5)	174 (1.0)	65 (2.9)	23 (4.2)	22 (3.2)	23 (5.6)	22 (3.9)	<.001
Self‐reported comorbidities (%)								
Diabetes	489 (2.2)	290 (1.6)	77 (3.4)	38 (7.0)	26 (3.8)	23 (5.6)	35 (6.2)	<.001
Stroke	303 (1.4)	143 (0.8)	63 (2.8)	40 (7.4)	18 (2.7)	13 (3.2)	26 (4.6)	<.001
Cancer	1185 (5.4)	883 (5.0)	136 (6.0)	42 (7.7)	57 (8.4)	31 (7.6)	36 (6.4)	<.001
Heart attack	693 (3.1)	262 (1.5)	162 (7.2)	93 (17.2)	72 (10.6)	47 (11.5)	57 (10.1)	<.001
Mean plasma fibrinogen (g/L) (SD)	2.9 (1.0)	2.9 (1.0)	3.1 (0.9)	3.2 (0.9)	3.1 (0.9)	3.1 (0.9)	3.2 (0.9)	<.001

*Note*: Values presented are mean (SD) for normally distributed, continuous data, median (IQR) for non‐normally distributed, continuous data and number (%) for categorical data. Total ACB was calculated with the formula (number of class 1 anticholinergics) + (number of class 2 anticholinergics × 2) + (number of class 3 anticholinergics × 3).

Abbreviations: ACB, anticholinergic cognitive burden; BMI, body mass index; NSAID, nonsteroidal anti‐inflammatory drugs.

There were significant differences between ACB groups for all variables considered except fruit and vegetable consumption. People in the higher ACB groups were older, had higher BMI and total cholesterol level, and a lower level of physical activity. NSAIDs and lipid‐lowering drug usage was more prevalent in higher ACB groups. In terms of comorbidities, high ACB score was associated with a greater percentage of individuals with a prior history of stroke, cancer, diabetes and myocardial infarction. Significantly higher mean fibrinogen and median CRP were observed in higher ACB groups compared to the ACB = 0 group.

Supporting Information Table S2 (online resources) details the characteristics of participants in 2HC, stratified by the ACB groups. There were 5101 participants, of which 61.5% were women and the mean (SD) age was 63.1 years (8.9). The mean (SD) TNF‐α was 2.0 pg/mL (0.8) whilst the median (IQR) IL‐6 was 0.6 (0.5‐0.9). People in the higher ACB groups were older, had higher BMI and were less likely to be active. There were no significant sex differences between ACB groups or in terms of fruit and vegetable consumption. There were higher proportions of NSAID and lipid‐lowering drug usage in higher ACB groups. With regard to self‐reported illnesses, high ACB was associated with a greater percentage of individuals with a prior history of stroke, diabetes and myocardial infarction. However, there were no significant differences between ACB groups in terms of cancer prevalence. There was a significant difference between ACB groups in terms of both TNF‐α and IL‐6: the levels of these inflammatory markers were higher in participants in higher ACB groups compared to the ACB = 0 group.

### 3.2 Cross‐sectional analyses

Table 2 shows that when compared to the reference category of ACB = 0, ACB = 1 to ACB ≥ 4 were associated with increases in all inflammatory markers. In fibrinogen (g/L) (95% confidence interval (CI); *P* value) an increase by 0.118 (0.074, 0.162; *P* < .001) and 0.134 (0.070, 0.199; *P* < .001) was seen. CRP was associated with the highest increase when the ACB score was compared to ACB = 0. There was an increase in CRP (mg/L) (95% CI; *P* value) by 0.765 (0.447, 1.083; *P* < .001) and 1.175 (0.715, 1.634; *P* < .001) when ACB = 1 and ACB ≥ 4, respectively. In the same way, when compared to ACB = 0, ACB = 1 and ACB ≥ 4 were associated with 0.073 (0.005, 0.141; *P* = .035) and 0.593 (0.254, 0.932; *P* = .001) increases in TNF‐α (pg/mL), respectively. However, IL‐6 (pg/mL) showed a decrease of 0.040 (−0.260, 0.180; *P* = .724) when comparing the reference category to ACB = 1. This was due to the reduced sample size. IL‐6 showed an increase of 0.593 pg/mL (0.254, 0.932; *P* = .001) when ACB ≥ 4 was compared to the reference category. These percentage increases get larger as the ACB score grows, therefore the relationship between ACB and inflammatory marker is a linear one. This is a cross‐sectional relationship due to inflammatory markers being collected at the same health check at which the ACB scores were collected.

**TABLE 2:** Results of multivariable linear regressions evaluating the cross‐sectional relationship between ACB scores at baseline and levels of inflammatory markers measured at the first health check when compared to ACB = 0

ACB score 95% CI *P* value	Δfibrinogen (g/L) (1HC)	ΔCRP (mg/L) (1HC)	ΔIL‐6 (pg/mL) (2HC)	ΔTNF‐α (pg/mL) (2HC)
ACB = 0	Ref	Ref	Ref	Ref
ACB = 1	0.118 (0.074, 0.162) *P* < .001	0.765 (0.447, 1.083) *P* < .001	−0.040 (−0.260,0.180) *P* = .724	0.073 (0.005, 0.141) *P* = .035
ACB = 2	0.090 (0.005, 0.175) *P* = .039	0.658 (0.040, 1.277) *P* = .037	0.056 (−0.388, 0.500) *P* = .805	0.125 (−0.12, 0.261) *P* = .074
ACB = 3	0.094 (0.018, 0.170) *P* = .016	0.903 (0.368, 1.439) *P* = .001	0.221 (−0.185, 0.627) *P* = .286	0.030 (−0.95, 0.154) *P* = .642
ACB ≥ 4	0.134 (0.070, 0.199) *P* < .001	1.175 (0.715, 1.634) *P* < .001	0.593 (0.254, 0.932) *P* = .001	0.137 (0.033, 0.241) *P* = .010

*Note*: This shows the increase in inflammatory marker when the ACB scores 1 to ≥4 were compared to the reference category, ACB = 0. Inflammatory markers fibrinogen and CRP were collected in the first health check and TNF‐α and IL‐6 were collected in the second health check. To demonstrate a cross‐sectional relationship, the exposure for fibrinogen and CRP was baseline data from the first health check, whilst for IL‐6 and TNF‐α the exposure was participants' data from the second health check. This table shows a multivariable model adjusted for age, sex, lifestyle factors (BMI, alcohol consumption smoking status, total fruit and vegetables consumed, and physical activity), total cholesterol, medications (lipid‐lowering drugs, NSAIDs) and self‐reported comorbidities (stroke, cancer, heart attack and diabetes).

Abbreviations: 1HC, first health check; 2HC, second health check; ACB, anticholinergic cognitive burden; CI, confidence interval; CRP, C‐reactive protein; IL‐6, interleukin 6; Ref, reference category; TNF‐α, tumour necrosis factor alpha.

Table 4 shows the results of linear regression analysis for the cross‐sectional relationship between baseline ACB groups and inflammatory markers. All the adjusted models showed that a unit increase in the ACB score was associated with a significant increase in all the inflammatory markers. After complete multivariable adjustment (model 4), a unit increase in ACB score was associated with an increase of fibrinogen of 0.035 g/L (0.006; *P* < .001), CRP of 0.284 mg/L (0.044; *P* < .001) (in 1HC), TNF‐α of 0.031 pg/mL (0.010; *P* = .002) and IL‐6 of 0.112 pg/mL (0.033; *P* = .001) (in 2HC), respectively.

### 3.3 Prospective analysis

Table 3 shows the prospective relationship that describes the association between the inflammatory marker collected in the HC2 compared to the ACB score collected in the HC1. The reference category of ACB = 0 when compared to ACB = 1 and ACB ≥ 4 was associated with increases in IL‐6 (pg/mL) by 0.116 (−0.112, 0.344; *P* = .319) and 0.019 (−0.323, 0.361; *P* = .915), respectively, and in TNF‐α (pg/mL) by 0.012 (−0.068, 0.093; *P* = .762) and 0.202 (0.81, 0.323; *P* = .001), respectively.

**TABLE 3:** Results of multivariable linear regressions evaluating the prospective relationship between ACB scores at baseline and levels of inflammatory markers measured at the second health check when compared to ACB = 0

ACB score 95% CI *P* value	ΔIL‐6 (pg/mL) (2HC)	ΔTNF‐α (pg/mL) (2HC)
ACB = 0	Ref	Ref
ACB = 1	0.116 (−0.112, 0.344) *P* = .319	0.012 (−0.068, 0.093) *P* = .762
ACB = 2	−0.109 (−0.581, 0.362) *P* = .649	0.159 (−0.007,0.326) *P* = .061
ACB = 3	0.129 (−0.267, 0.525) *P* = .523	−0.084 (−0.224, 0.056) *P* = .241
ACB ≥ 4	0.019 (−0.323, 0.361) *P* = .915	0.202 (0.81, 0.323) *P* = .001

*Note*: This shows the increase in inflammatory marker when the ACB scores 1 to ≥4 were compared to the reference category, ACB = 0. The exposure was baseline data from the first health check to demonstrate a prospective relationship because TNF‐α and IL‐6 were the only inflammatory markers collected in the second health check. This table shows a multivariable model adjusted for age, sex, lifestyle factors (BMI, alcohol consumption smoking status, total fruit and vegetables consumed, and physical activity), total cholesterol, medications (lipid‐lowering drugs, NSAIDs) and self‐reported comorbidities (stroke, cancer, heart attack and diabetes).

Abbreviations: 1HC, first health check; 2HC, second health check; ACB, anticholinergic cognitive burden; CI, confidence interval; IL‐6, interleukin 6; Ref, reference category; TNF‐α, tumour necrosis factor alpha.

**TABLE 4:** Results of multivariable linear regressions evaluating the cross‐sectional relationship between unit increases in ACB scores at baseline and levels of inflammatory markers measured at the first health check

Inflammatory marker	Model	*β* value change in inflammatory marker for 1‐point increase in ACB (95% CI)	*P* value
Fibrinogen (g/L) (1HC)	A	0.074 (0.063, 0.086)	<.001
	B	0.046 (0.035, 0.058)	<.001
	C	0.037 (0.025, 0.049)	<.001
	D	0.035 (0.023, 0.047)	<.001
CRP (mg/L) (1HC)	A	0.465 (0.381, 0.549)	<.001
	B	0.372 (0.287, 0.456)	<.001
	C	0.304 (0.220, 0.389)	<.001
	D	0.284 (0.198, 0.369)	<.001
IL‐6 (pg/mL) (2HC)	A	0.129 (0.068, 0.190)	<.001
	B	0.113 (0.051, 0.174)	<.001
	C	0.107 (0.045, 0.169)	.001
	D	0.112 (0.048, 0.175)	.001
TNF‐α (pg/mL) (2HC)	A	0.065 (0.045, 0.084)	<.001
	B	0.042 (0.023, 0.061)	<.001
	C	0.037 (0.018, 0.056)	<.001
	D	0.031 (0.011, 0.050)	.002

*Note*: This is the linear regression analysis of inflammatory markers and ACB score. It shows how a unit increase in ACB score is associated with the inflammatory markers. The inflammatory markers fibrinogen and CRP were collected in the first health check and TNF‐α and IL‐6 were collected in the second health check. To demonstrate a cross‐sectional relationship, the exposure for fibrinogen and CRP was baseline data from the first health check whilst for IL‐6 and TNF‐α the exposure was participants' data from the second health check. The following models adjust for the ACB groups and inflammatory marker: A = univariate model, B = age and sex, C = age, sex and lifestyle factors (BMI, alcohol consumption smoking status, total fruit and vegetables consumed, and physical activity), D = age, sex, lifestyle factors, total cholesterol, medications (lipid‐lowering drugs, NSAIDs) and self‐reported comorbidities (stroke, cancer, heart attack and diabetes).

Abbreviations: 1HC, first health check; 2HC, second health check; ACB, anticholinergic cognitive burden; CI, confidence interval; CRP, C‐reactive protein; IL‐6, interleukin 6; TNF‐α, tumour necrosis factor alpha.

**TABLE 5:** Results of multivariable linear regressions evaluating the prospective relationship between unit increases in ACB scores at baseline and levels of inflammatory markers measured at the second health check

Inflammatory marker	Model	*β* value change in inflammatory marker for 1 point increase in ACB (95% CI)	*P* value
IL‐6 (pg/mL) (2HC)	A	0.098 (0.030, 0.166)	.005
	B	0.078 (0.009, 0.146)	.026
	C	0.079 (0.013, 0.145)	.020
	D	0.076 (0.008, 0.144)	.029
TNF‐α (pg/mL) (2HC)	A	0.064 (0.043, 0.085)	<.001
	B	0.040 (0.019, 0.061)	<.001
	C	0.036 (0.015, 0.058)	.001
	D	0.028 (0.006, 0.050)	.013

*Note*: This is the linear regression analysis for inflammatory markers and ACB score. It shows how a unit increase in ACB score is associated with the inflammatory markers. The exposure was baseline data from the first health check to demonstrate a prospective relationship because TNF‐α and IL‐6 were the only inflammatory markers collected in the second health check.

The following models adjust for the ACB groups and inflammatory marker: A = univariate model, B = age and sex, C = age, sex and lifestyle factors (BMI, alcohol consumption, smoking status, total fruits and vegetables consumed and physical activity), D = age, sex, lifestyle factors, total cholesterol, medications (lipid lowering drugs, NSAIDs), co‐morbidities (stroke, cancer, heart attack and diabetes).

Abbreviations: 1HC, first health check; 2HC, second health check; ACB, anticholinergic cognitive burden; CI, confidence interval; IL‐6, interleukin 6; TNF‐α, tumour necrosis factor alpha.

Table 5 shows the results of the prospective analyses for a prospective relationship between 1HC ACB and inflammatory markers measured at the 2HC. All models revealed a statistically significant association between ACB score at 1HC and increase in the circulating levels of inflammatory markers measured at 2HC. On full multivariable adjustment, a unit increase in the ACB score was associated with a significant increase in TNF‐α and IL‐6 of 0.028 pg/mL (0.011; *P* = .013) and 0.076 pg/mL (0.035; *P* = .029), respectively.

## 4 DISCUSSION

We describe for the first time the association between anticholinergic burden and inflammation at a population level. Cross‐sectionally, increases in ACB scores were independently associated with significant increases in fibrinogen, CRP, TNF‐α and IL‐6. Prospectively, increases in ACB scores were independently associated with increases in TNF‐α and IL‐6. To the best of our knowledge, this is the first report which provides a possible mechanistic link through inflammatory pathways between anticholinergic medications and adverse longer‐term outcomes at a population level.

Increases in such inflammatory markers in the long term can lead to adverse outcomes. The meta‐analysis by Kaptoge et al^20^ found a significant 17% increase (risk ratio (RR) 1.17 [1.09‐1.25]) and 25% increase (RR 1.25 [1.19‐1.32]) in incident coronary heart disease (CHD)/nonfatal MI associated with a 1 SD increase in TNF‐α and IL‐6, respectively. Our study suggests that 1‐ and 3‐point ACB increases would translate into 0.646% and ~2% relative risk increases in TNF‐α‐mediated incident CHD/nonfatal MI, respectively.^20^ Furthermore, our findings also suggest that 1‐ and 3‐point ACB increases would translate into 1.13% and 3.39% relative risk increases in IL‐6‐mediated incident CHD/nonfatal MI, respectively.^20^ Prospectively, a 1‐point increase in ACB would translate into a 0.595% and 0.75% increase in the relative risk of TNF‐α‐mediated incident CHD/nonfatal MI and IL‐6‐mediated incident CHD/nonfatal MI, respectively.^20^

The meta‐analysis by Yano et al^21^ established that a 1 SD increase in fibrinogen was associated with a 30% increase (RR 1.3 [1.2‐1.4]) in all‐cause mortality, a 20% increase (RR 1.2 [1.1‐1.4]) in cardiovascular disease and a 30% increase (RR1.3 [1.2 to 1.5]) in cancer. Cross‐sectionally, our study found a 1‐point increase in ACB would translate into a 1.05% increase in the relative risk of all‐cause mortality, a 0.7% increase in the relative risk of cardiovascular disease and a 1.05% increase in the relative risk of cancer.^21^ In addition, the Emerging Risk Factors Collaboration^22^ also defined an increase in relative risk of CHD and ischaemic stroke associated with CRP. Our findings revealed that an increase in ACB would translate into an increase in the relative risk of CHD and ischaemic stroke.

The results of our study alongside previous investigations linking markers of chronic inflammation with a variety of adverse incident outcomes allow, for the first time, the quantification of the contribution of inflammation in mediating the relationship between ACB and adverse outcomes. The inflammatory reflex may mediate the described relationships between anticholinergic burden and adverse outcomes. We hypothesised that an increased anticholinergic burden may lead to raised inflammatory markers due to the inflammatory reflex.^16^ This mechanism, also known as the cholinergic anti‐inflammatory pathway, suggests that the vagus nerve, a part of the parasympathetic nervous system, has an anti‐inflammatory role. Central vagus nerve stimulation downregulates inflammatory responses and thus we hypothesised that increased anticholinergic burden leads to increased inflammation by blocking central muscarinic‐dependent vagal activation.^17^

Based on literature, our findings can be extrapolated that a 3‐point increase in ACB‐related chronic inflammation can be linked to an increase in relative risk of up to ~4% in cancer, cardiovascular disease and mortality. Furthermore, a previous study^7^ including participants from the EPIC‐Norfolk cohort found that people with a total ACB ≥ 3 from medications had a relative risk increase of mortality of 83% (hazard ratio (HR) 1.83 [1.53‐2.20]) and a 117% increase in incident CVD. The increased risk of up to 3.39% in incident CHD/nonfatal MI after a 3‐point increase in ACB inferred from the study by Kaptoge et al^20^ would account for only 2.9% of the entire ACB ≥ 3‐associated excess risk of incident CVD.

Indeed, there may be plausible mechanisms other than chronic inflammation that may mediate the association between ACB from medications and adverse outcomes. Anticholinergic drugs have been found to suppress the parasympathetic control of heart rate, which is linked to an increased incidence of myocardial ischemia and tachyarrythmias that are known to increase the risk of embolic strokes and sudden cardiac death.^23^ Anticholinergic medications can also lead to ischaemia due to their pro‐ischaemic properties.^24^ In addition, the arterial baroreflex is an important mechanism that protects against strokes.^25^ The anticholinergic medications disrupt vagal nerve activity, which is involved in the activation of arterial baroreflex, therefore reducing its protective effects.^25^

Our study has several strengths. It benefited from using data from a large, prospective population‐based study. We were able to delineate both the cross‐sectional and prospective associations between ACB and markers of inflammation. The data were also prospectively collected, minimising recall bias. Additionally, a well‐validated ACB score was used, and we were able to control for a variety of sociodemographic and lifestyle factors, comorbidities and relevant medications.

We acknowledge some limitations. As a volunteer study with long‐term follow‐up, a degree of healthy volunteer bias is possible. However, the baseline characteristics of the EPIC‐Norfolk participants are similar to other UK representative population samples.^18^ The participants in this study were almost completely (>99%) White British, but it is unlikely biological mechanisms will be hugely different from other ethnicities. Potential confounders adjusted were measured at baseline, and it is possible that these may vary during the follow‐up period. Although we were able to calculate the total ACB score, we were not able to identify particular drugs and the dosages that are associated to adverse outcomes. In addition, as the ACB score was calculated at baseline, we were unable to account for any changes in ACB score during the follow‐up period for prospective analysis. Nevertheless, it is likely that individuals would either maintain similar anticholinergic exposure or be exposed to increasing anticholinergic burden during follow‐up because the use of medications with anticholinergic properties would increase as participants age and accrue more disease burden and increasing polypharmacy. However, both cross‐sectional and prospective analyses results for TNF‐α and IL‐6 were consistent.

This study, along with future robust experimental studies, may change the anticholinergic burden paradigm by recognizing the chronic inflammatory state associated with anticholinergic medications as a therapeutic target in the era of targeted immune therapy for cardiovascular prevention. The landmark CANTOS trial used canakinumab to target the interleukin‐1β innate immunity pathway, leading to a significantly lower incidence of recurrent cardiovascular events.^26^ Furthermore, colchicine has been shown to be effective at preventing major adverse cardiac events after a myocardial infarction^27^ and preventing cardiovascular events in patients with recent myocardial infarction^28^ and stable coronary disease.^29^, ^30^ Future studies should also explore whether systematic attempts to reduce the anticholinergic burden reduce inflammation and, consequently, the risk of such adverse outcomes.

In conclusion, using data from a large‐scale prospective cohort study from the UK, we determined the cross‐sectional and prospective associations between the anticholinergic burden and inflammatory markers. Higher anticholinergic burden is significantly associated with higher inflammatory markers both cross‐sectionally and prospectively after multivariable adjustment. Furthermore, we underlined how chronic inflammation may be a previously unrecognized potential mechanism for the observed association between anticholinergic burden and adverse outcomes.

## COMPETING INTERESTS

The authors have no conflicts of interest to declare that are relevant to the content of this article.

## CONTRIBUTORS

R.S., T.A.P. and P.K.M. conceived the study. R.S. performed the literature search and data analysis, and wrote the first draft of the paper. R.S., T.A.P. and P.K.M. verified the data. N.J.W. and K.‐T.K. are the PIs of the EPIC‐Norfolk cohort. All authors commented on previous versions of the manuscript and approved the final manuscript. P.K.M. is the guarantor.

## ETHICS APPROVAL

The Norwich Ethics Committee approved the study in view of the retrospective nature of the study.

## CONSENT TO PARTICIPATE AND PUBLISH

Informed consent was obtained from all individual participants included in the study.

## Supporting information


**Supporting Information Table S1** Baseline sample characteristics for 17 678 men and women whose CRP level was measured in the EPIC‐Norfolk cohort (first health check) according to the total anticholinergic burden score groups
**Supporting Information Table S2** Characteristics from second health check for 5101 men and women whose TNF‐α and IL‐6 levels were measured in the EPIC‐Norfolk cohort according to the total anticholinergic burden score groups

## Data Availability

Data available on request to EPIC‐Norfolk Steering Committee for their approval.
