# Supplementary material for: Higher anticholinergic burden from medications is associated with significant increase in markers of inflammation in the EPIC‐Norfolk prospective population‐based cohort study
Source: Br J Clin Pharmacol. 2022 Feb 27;88(7):3297–306. doi: 10.1111/bcp.15261 (PMC9373850; doi:10.1111/bcp.15261)
Supplement: Supplementary file 1 — Supporting Information Table S1 Baseline sample characteristics for 17 678 men and women whose CRP level was measured in the EPIC‐Norfolk cohort (first health check) according to the total anticholinergic burden score groups Supporting Information Table S2 Characteristics from second health check for 5101 men and women whose TNF‐α and IL‐6 levels were measured in the EPIC‐Norfolk cohort according to the total anticholinergic burden score groups [file BCP-88-3297-s001.docx]

**Online Resources**

**Table S1** Baseline sample characteristics for 17,678 men and women whose CRP level was measured in the EPIC-Norfolk cohort (first health check) according to the total anticholinergic burden score groups.

|  | **All** | **ACB score 0 group** | **ACB score 1 group** | **ACB score 2 group** | **ACB score 3 group** | **ACB score 4 group** | **ACB score ≥5 group** | ***p* value** |
| --- | --- | --- | --- | --- | --- | --- | --- | --- |
|  | N= 17678 | N=14161 | N=1758 | N=422 | N=560 | N=332 | N=445 |  |
| **Mean age (years) (SD)** | 59.1 (9.1) | 58.2 (9.0) | 62.4 (8.8) | 65.4 (8.2) | 62.1 (9.1) | 64.8 (8.2) | 62.5 (9.0) | <0.001 |
| **Sex (%)** |  |  |  |  |  |  |  | <0.001 |
| **Men** | 7942 (44.9) | 6343 (44.8) | 805 (45.8) | 232 (55.0) | 226 (40.4) | 168 (50.6) | 168 (37.8) |  |
| **Women** | 9736 (55.1) | 7818 (55.2) | 953 (54.2) | 190 (45.0) | 334 (59.6) | 164 (49.4) | 277 (62.2) |  |
| **Mean BMI (kg/m^2)**  **(SD)** | 26.2 (3.8) | 26.1 (3.7) | 26.8 (4.1) | 27.0 (4.0) | 26.6 (4.1) | 27.0 (3.9) | 27.2 (4.1) | <0.001 |
| **Smoking status (%)** |  |  |  |  |  |  |  | <0.001 |
| **Current** | 1959 (11.1) | 1590 (11.2) | 168 (9.6) | 44 (10.4) | 65 (11.6) | 23 (6.9) | 69 (15.5) |  |
| **Former** | 7368 (41.7) | 5704 (40.3) | 826 (47.0) | 218 (51.7) | 253 (45.2) | 166 (50.0) | 201 (45.2) |  |
| **Never** | 8351 (47.2) | 6867 (48.5) | 764 (43.5) | 160 (37.9) | 242 (43.2) | 143 (43.1) | 175 (39.3) |  |
| **Median alcohol consumption (units/week)**  **(IQR)** | 3.5 (1.0-10.0) | 4.0 (1.0-10.0) | 2.5 (1.0- 9.0) | 2.5 (1.0- 9.0) | 2.5 (0.5- 8.5) | 2.5 (1.0-9.5) | 2.0 (0.5-6.8) | <0.001 |
| **Physical activity (%)** |  |  |  |  |  |  |  | <0.001 |
| **Inactive** | 5216 (29.5) | 3791 (26.8) | 684 (38.9) | 196 (46.4) | 207 (37.0) | 149 (44.9) | 189 (42.5) |  |
| **Moderately inactive** | 5096 (28.8) | 4111 (29.0) | 489 (27.8) | 108 (25.6) | 172 (30.7) | 89 (26.8) | 127 (28.5) |  |
| **Moderately active** | 4053 (22.9) | 3410 (24.1) | 344 (19.6) | 61 (14.5) | 108 (19.3) | 42 (12.7) | 88 (19.8) |  |
| **Active** | 3313 (18.7) | 2849 (20.1) | 241 (13.7) | 57 (13.5) | 73 (13.0) | 52 (15.7) | 41 (9.2) |  |
| **Mean total cholesterol**  **(mmol/l)**  **(SD)** | 6.2 (1.2) | 6.1 (1.1) | 6.3 (1.2) | 6.3 (1.3) | 6.3 (1.2) | 6.4 (1.3) | 6.4 (1.3) | <0.001 |
| **Mean total fruits and vegetables consumed**  **(g/day)**  **(SD)** | 511.2 (248.0) | 509.5 (246.1) | 521.3 (262.8) | 503.0 (250.0) | 518.4 (247.1) | 512.4 (221.5) | 522.5 (264.4) | 0.17 |
| **NSAID (%)** | 2592 (14.7) | 1700 (12.0) | 400 (22.8) | 129 (30.6) | 153 (27.3) | 91 (27.4) | 119 (26.7) | <0.001 |
| **Lipid lowering drugs (%)** | 256 (1.4) | 135 (1.0) | 49 (2.8) | 18 (4.3) | 17 (3.0) | 18 (5.4) | 19 (4.3) | <0.001 |
| **Self-reported co-morbidities (%)** |  |  |  |  |  |  |  |  |
| **Diabetes** | 384 (2.2) | 230 (1.6) | 63 (3.6) | 28 (6.6) | 23 (4.1) | 18 (5.4) | 22 (4.9) | <0.001 |
| **Stroke** | 255 (1.4) | 125 (0.9) | 56 (3.2) | 30 (7.1) | 13 (2.3) | 12 (3.6) | 19 (4.3) | <0.001 |
| **Cancer** | 923 (5.2) | 686 (4.8) | 107 (6.1) | 29 (6.9) | 47 (8.4) | 24 (7.2) | 30 (6.7) | <0.001 |
| **Heart attack** | 583 (3.3) | 225 (1.6) | 131 (7.5) | 71 (16.8) | 65 (11.6) | 41 (12.3) | 50 (11.2) | <0.001 |
| **Median CRP (mg/l) (IQR)** | 1.5 (0.7-3.3) | 1.4 (0.7-3.0) | 2.1 (1.0-4.4) | 2.4 (1.2-4.7) | 2.0 (1.0-4.6) | 2.3 (1.2-4.7) | 2.5 (1.2-5.4) | <0.001 |

Note: Values presented are mean (SD) for normally distributed, continuous data, median (IQR) for non- normally distributed, continuous data and number (%) for categorical data. Total ACB was calculated with the formula of ((number of class 1 anticholinergics) + (number of class 2 anticholinergics × 2) + (number of class 3 anticholinergics × 3)).

Abbreviations: ACB, anticholinergic cognitive burden; BMI, body mass index; NSAID, non-steroidal anti-inflammatory drugs; CRP, C-reactive protein.

**Table S2** Characteristics from 2^nd^ health-check for 5,101 men and women whose TNF-α and IL-6 levels were measured in the EPIC-Norfolk cohort according to the total anticholinergic burden score groups.

|  | **All** | **ACB score 0 group** | **ACB score 1 group** | **ACB score 2 group** | **ACB score 3 group** | **ACB score 4 group** | **ACB score ≥5 group** | ***p* value** |
| --- | --- | --- | --- | --- | --- | --- | --- | --- |
|  | N=5101 | N=3969 | N=608 | N=133 | N=157 | N=79 | N=155 |  |
| **Mean age (years) (SD)** | 63.1(8.9) | 62.2(8.8) | 65.8(8.5) | 68.6(8.5) | 65.6(8.3) | 68.7(8.3) | 65.6(8.4) | <0.001 |
| **Sex (%)** |  |  |  |  |  |  |  | 0.135 |
| **Men** | 1963(38.5) | 1502(37.8) | 247(40.6) | 61(45.9) | 57(36.3) | 38(48.1) | 58(37.4) |  |
| **Women** | 3138(61.5) | 2467(62.2) | 361(59.4) | 72(54.1) | 100(63.7) | 41(51.9) | 97(62.6) |  |
| **Mean BMI (kg/m^2)**  **(SD)** | 26.6(3.8) | 26.4(3.8) | 27.2(4.1) | 27.5(4.3) | 27.1(4.0) | 27.4(4.2) | 27.8(3.8) | <0.001 |
| **Smoking status (%)** |  |  |  |  |  |  |  | 0.002 |
| **Current** | 351(6.9) | 276(7.0) | 36(5.9) | 6(4.5) | 12(7.6) | 2(2.5) | 19(12.3) |  |
| **Former** | 2165(42.4) | 1633(41.1) | 281(46.2) | 58(43.6) | 81(51.6) | 40(50.6) | 72(46.5) |  |
| **Never** | 2585(50.7) | 2060(51.9) | 291(47.9) | 69(51.9) | 64(40.8) | 37(46.8) | 64(41.3) |  |
| **Median alcohol consumption (units/week)**  **(IQR)** | 3.0(1.0-9.0) | 3.0(1.0-9.0) | 2.5(1.0-9.0) | 3.0(0.5-8.5) | 3.0(1.0-8.5) | 3.0(1.0-9.0) | 2.0(0.5-9.0) | 0.046 |
| **Physical activity (%)** |  |  |  |  |  |  |  | <0.001 |
| **Inactive** | 378(7.4) | 260(6.6) | 49(8.1) | 21(15.8) | 18(11.5) | 7(8.9) | 23(14.8) |  |
| **Moderately inactive** | 1867(1238) | 1412(35.6) | 247(40.6) | 48(36.1) | 70(44.6) | 27(34.2) | 63(40.6) |  |
| **Moderately active** | 1238(24.3) | 979(24.7) | 142(23.4) | 29(21.8) | 35(22.3) | 20(25.3) | 33(21.3) |  |
| **Active** | 1618(31.7) | 1318(33.2) | 170(28.0) | 35(26.3) | 34(21.7) | 25(31.6) | 36(23.2) |  |
| **Mean total cholesterol**  **(mmol/l)**  **(SD)** | 6.1(1.2) | 6.1(1.2) | 6.1(1.1) | 6.0(1.3) | 6.1(1.2) | 6.1(1.0) | 6.4(1.3) | 0.008 |
| **Mean total fruits and vegetables consumed**  **(g/day)**  **(SD)** | 538.6(254.4) | 540.0(257.5) | 533.6(235.4) | 501.5(205.2) | 534.9(270.0) | 592.7(248.2) | 531.9(270.6) | 0.895 |
| **NSAID (%)** | 1100(21.6) | 690(17.4) | 199(32.7) | 61(45.9) | 60(38.2) | 32(40.5) | 58(37.4) | <0.001 |
| **Lipid lowering drugs (%)** | 275(5.4) | 141(3.6) | 66(10.9) | 23(17.3) | 20(12.7) | 10(12.7) | 15(9.7) | <0.001 |
| **Self-reported co-morbidities(%)** |  |  |  |  |  |  |  |  |
| **Diabetes** | 161(3.2) | 102(2.6) | 22(3.6) | 10(7.5) | 10(6.4) | 10(12.7) | 7(4.5) | <0.001 |
| **Stroke** | 135(2.6) | 57(1.4) | 31(5.1) | 15(11.3) | 16(10.2) | 5(6.3) | 11(7.1) | <0.001 |
| **Cancer** | 371(7.3) | 276(7.0) | 48(7.9) | 14(10.5) | 12(7.6) | 7(8.9) | 14(9.0) | 0.539 |
| **Heart attack** | 157(3.1) | 46(1.2) | 52(8.6) | 19(14.3) | 11(7.0) | 11(13.9) | 18(11.6) | <0.001 |
| **Mean TNF-α (pg/ml) (SD)** | 2.0(0.8) | 2.0(0.8) | 2.1(0.9) | 2.3(1.0) | 2.1(0.7) | 2.1(0.7) | 2.3(1.1) | <0.001 |
| **Median IL-6 (pg/ml) (IQR)** | 0.6(0.5-0.9) | 0.6(0.5-0.9) | 0.7(0.5-1.0) | 0.8(0.5-1.1) | 0.7(0.5-1.0) | 0.8(0.6-1.1) | 0.8(0.56-1.2) | <0.001 |

Note: Values presented are mean (SD) for normally distributed, continuous data, median (IQR) for non- normally distributed, continuous data and number (%) for categorical data. Total ACB was calculated with the formula of ((number of class 1 anticholinergics) + (number of class 2 anticholinergics × 2) + (number of class 3 anticholinergics × 3)).

Abbreviations: ACB, anticholinergic cognitive burden; BMI, body mass index; NSAID, non-steroidal anti-inflammatory drugs; TNF-α, tumour necrosis factor alpha; IL-6, interleukin 6.
